# Barriers and enablers to the implementation of perioperative hypothermia prevention practices from the perspectives of the multidisciplinary team: a qualitative study using the Theoretical Domains Framework

**DOI:** 10.2147/JMDH.S209687

**Published:** 2019-05-29

**Authors:** Judy Munday, Alana Delaforce, Gillian Forbes, Samantha Keogh

**Affiliations:** 1 School of Nursing and Institute of Health and Biomedical Innovation, Queensland University of Technology, Kelvin Grove, QLD 4059, Australia; 2 Department of Health and Nursing Science, University of Agder, Grimstad, Norway; 3 Clinical Governance Unit, Mater Health, South Brisbane, QLD 4101, Australia; 4 Department of Clinical, Educational and Health Psychology, UCL Centre for Behaviour Change, London WC1E 6BT, UK

**Keywords:** perioperative hypothermia, temperature management, Theoretical Domains Framework, multidisciplinary, COM-B, behaviour change wheel

## Abstract

**Purpose:** Inadvertent perioperative hypothermia is a significant problem for surgical patients globally, and is associated with many detrimental side-effects. Despite the availability of rigorously developed international evidence-based guidelines for prevention, a high incidence of this complication persists. This qualitative study aims to identify and examine the domains which act as barriers and enablers to perioperative hypothermia prevention practices, from the perspectives of the key healthcare professionals involved with perioperative temperature management.

**Methods:** A qualitative study employing semi-structured interviews was utilized. A purposive sample of key stakeholders involved in perioperative temperature management, including perioperative nurses, anesthetists, surgeons, and perioperative managers, were recruited via email. The interview guide was developed in reference to the Theoretical Domains Framework. All interviews were recorded, de-identified, transcribed, and coded. Belief statements were generated within each domain, and a frequency score generated for each belief. Finally, the domains were mapped to the COM-B model of the Behavior Change Wheel to develop recommendations for future interventions.

**Results:** Twelve participants were included including eight nurses, two surgeons, and two anesthetists. Eleven key theoretical domains that influence the uptake of perioperative hypothermia practices were identified: knowledge; skills; social/professional role and identity; beliefs about capabilities; optimism; beliefs about consequences; reinforcement; goals; memory, attention, and decision processes; environmental context and resources; social influence. Suggested intervention strategies include training, reminder systems, audit, and feedback, organizational support to resolve lack of control of ambient temperature, as well as provision of accurate temperature measurement devices.

**Conclusion:** Future interventions to address the key behavioral domains and improve perioperative hypothermia prevention need to be evaluated in the context of feasibility, effectiveness, safety, acceptability, and cost by the target users. All suggested intervention strategies need to take a team-based, multi-modal approach, as this is most likely to facilitate improvements in perioperative hypothermia prevention.

## Introduction

Inadvertent perioperative hypothermia is a significant problem for surgical patients globally,^1^ and is associated with a range of adverse side-effects, including increased blood loss,^2^ increased wound infection rates,^3^ decreased immune function,^4^ shivering, prolonged duration of medications including muscle relaxants, increased Post Anesthetic Care Unit (PACU) stay and overall hospital stay,^3^ and patient discomfort. This leads to an impact on the efficiency of perioperative services, and increased costs to the healthcare system.^5^ Perioperative hypothermia is preventable, however an incidence of perioperative hypothermia between 50–54% is reported in general adult surgical patients,^6^,^7^ and up to 80% in obstetric patients under spinal anesthesia.^8^ This is unacceptable given the available high level evidence base consisting of rigorously developed, and regularly reviewed international evidence-based guidelines which recommend the utilization of multi-faceted interventions to manage and prevent the condition.^1^,^9^,^10^ Most recently, in Australia, the Australian College of Perioperative Nurses (ACORN) have published guidance on the prevention of perioperative hypothermia: the Standard on the Management of Hypothermia in the Perioperative Environment.^11^ This recommends the identification of high risk patients, regular and consistent temperature monitoring at all stages of the perioperative pathway, the use of active warming measures, guidance on ambient temperature levels, and, importantly, clear communication regarding thermal care between all members of the perioperative team at all stages, and with patients and their carers. However, despite an abundance of primary research, synthesized evidence, and guidelines^1^,^9^,^10^,^12^,^13^ to promote the prevention of inadvertent perioperative hypothermia, a significant variation in practice remains, and high rates of perioperative hypothermia persist.

Safe perioperative care, including thermal care, requires collaboration between, and is the responsibility of, all members of the perioperative team – medical, non-nursing, and nursing - with management support also vital. Yet, the perioperative department has been – up until recently – under-investigated in relation to barriers and enablers to implementation of best practice guidelines.^14^ As well as recent guidelines,^11^ a collaboratively developed intervention bundle, based on evidence-based recommendations, has also been published in Australia,^15^ but did not result in a reduction of the incidence of perioperative hypothermia or specifically examine the barriers and enablers to uptake of recommendations. Implementation of care that aligns with evidence-based recommendations and bundles for perioperative hypothermia prevention may require understanding the barriers and enablers to changing behavior of multidisciplinary teams, and across multiple phases of the perioperative pathway. In addition, the level of change required to comply with guidelines and improve thermal care will depend upon the baseline level of thermal care already provided by healthcare facilities, as well as the level of adoption. Therefore, a pragmatic decision was made to identify the barriers and enablers to implementation using the Theoretical Domains Framework (TDF).^17^ A recent investigation utilized the TDF to identify factors that Canadian anesthetists perceived to influence perioperative temperature measurement (one component of perioperative hypothermia prevention).^16^ However, our study acknowledges the input that the wider perioperative team have in providing adequate thermal care, and investigates the wider domain of care that encompasses perioperative hypothermia prevention, rather than just temperature measurement alone.

The TDF is an overarching framework integrating a range of behavior change theories,^17^,^18^ and has been used to examine uptake of evidence in a variety of clinical settings.^16^,^19^–^22^ As extensively described in the literature, the framework includes 14 domains: 1) knowledge, 2) skills, 3) social/professional role and identify, 4) beliefs about capabilities, 5) optimism, 6) beliefs about consequences, 7) reinforcement, 8) intentions, 9) goals, 10) memory, attention, and decision processes, 11) environmental context and resources, 12) social influences, 13) emotion, and 14) behavioral regulation.^18^

The TDF, as part of the Behavior Change Wheel (BCW), guides a process for developing interventions and policy that specifically target deficits in three identified essential behavior change conditions: capability, opportunity, and motivation (referred to as the COM-B system).^23^ This taxonomy has been applied to inform the development of interventions and policies to improve delivery of healthcare in a variety of clinical settings.^20^ The TDF domains underpin the components of the COM-B system. In our study, the application of both the TDF and COM-B model assists us to develop intervention strategies to specifically target clinician-identified deficits in capability, opportunity, and motivation that influence perioperative hypothermia prevention. Therefore, this qualitative study aims to specifically examine the barriers and enablers that influence the uptake of temperature management practices to prevent perioperative hypothermia, from the perspectives of the key stakeholders involved with perioperative hypothermia prevention. Furthermore, the study aims to utilize the BCW to develop potential interventions to improve the implementation of perioperative hypothermia prevention.

## Methods

### Aim

This study aimed to investigate the barriers and enablers that influence the uptake of perioperative temperature management from multiple perspectives, namely, perioperative nurses, non-nursing or medical perioperative staff, anesthetic staff, surgical staff, and perioperative managers. Second, the study aimed to develop a potential intervention to improve implementation of perioperative hypothermia prevention practices.

### Design

A qualitative study design employing semi-structured interviews based on the TDF^17^ was used to explore enablers and barriers that influence perioperative temperature management amongst perioperative clinical staff. In addition, the COM-B model was utilized to assist in the development of potential intervention strategies to improve perioperative hypothermia prevention.^23^

### Setting, sample, and recruitment

Participants were recruited at a metropolitan, tertiary hospital in Brisbane, Australia. A purposive sample of key stakeholders involved in perioperative temperature management, including perioperative nurses, non-nursing or medical perioperative staff, anesthetic staff, surgical staff, and perioperative managers, were invited to participate via email. The study was also advertised via poster in the perioperative department. Informed consent was sought from individuals interested in participating. Low-risk ethical approval was obtained, prior to commencement, from the hospital human research ethics committee (HREC), and administrative approval was gained from the university HREC.

### Data collection

Individual semi-structured interviews were conducted utilizing an interview guide based on the TDF,^17^ with the intention of exploring the healthcare professional’s perspectives of perioperative hypothermia prevention practices. The interviews were conducted in a quiet interview room within the perioperative department, and at a time that suited the participant. Each question was developed to address each of the 14 theoretical domains, based on the literature and with input from a panel of experts (qualitative researcher, anesthetist, surgeon, perioperative nurse). However, unprompted issues were also explored during the interviews. Two researchers conducted all interviews (JM, AD), which were recorded, transcribed, and de-identified. Demographic data, including position, gender, number of years of experience, and duration of interview were collected at interview. Data saturation was assessed using the approach proposed by Francis et al,^24^ whereby no further interviews were conducted once no new information was observed to emerge.

### Data analysis

After transcription, two independent coders (JM, AD) analysed the textual data and assigned them into the 14 TDF domains. After data from two initial interviews was coded in this way, the coding was reviewed to establish a coding strategy for use with all remaining interview data. Discrepancies between reviewers were resolved via discussion. Second, thematic analysis was utilized to generate belief statements across the domains. The third coder (SK) reviewed the data analysis at this stage, followed by an expert in utilizing the TDF (GF). The identification of key domains likely to influence the implementation of perioperative hypothermia prevention was determined as per the three-pronged process utilized by Patey et al:^19^,^25^ the frequency of beliefs across the 12 interviews; the presence of conflicting beliefs within domains; perceived strength of the belief influencing the relevant behavior.^19^,^25^ Finally, the TDF domains were mapped to the capability (C), opportunity (O), and motivation (M) components, which form the “hub” of the BCW.^23^ Within each component, further sub-components were considered (Capability: psychological, physical; Opportunity: social, physical; Motivation: reflective, automatic (see Table 3),^23^ with the corresponding intervention function, Behavior Change Theory (BCT) taxonomy and individual BCT, to develop suggested intervention strategy examples.^26^ This study is reported in adherence to the Consolidated Criteria for Reporting Qualitative Research (COREQ) checklist.^27^

**Table 1:** TDF domains likely to influence the implementation of perioperative hypothermia prevention practices *denotes belief statements congruent with Boet et al.^16^

TDF domain	Specific belief	Example quotations	Frequency, out of 12
Knowledge	I am/am not aware that guidelines exist for the management of inadvertent hypothermia*	I am not aware of any official guidelines I must say, I don’t think I am aware of any guidelines per se, whether they be from the Australian College of Anesthetists or Surgeons or ACORN, I am sure they exist, but yeah. (A2) Not particularly any specific guidelines, I just know practices that improve the outcome and that’s what I do but I haven’t seen any written guidelines that have to be done every time. Are there such things? (SS5) Guidelines … I don’t … I am not aware of any Australian guidelines, there’s some British guidelines called NICE guidelines which are fairly strict. There is some auditing guidelines from the Australian anesthetic college, but they’re not actually guidelines on how to manage perioperative hypothermia, they are really just how to audit it. (A11)	11
	I/we have a lack of knowledge regarding the condition	Umm … it’s where their temperature drops, um and I know it can cause death, like well I know that’s malignant hyperthermia, is that the same or different? Okay, I don’t really know a lot then. (PN1) Again, it’s just getting to a number for discharge, I don’t think that the background knowledge of the actual importance of it being a certain range is there so I would just say that, because that was me you know when I still didn’t know all the ins and outs of it. (PN12)	9
	Education would assist me/us to increase our knowledge and awareness of managing perioperative hypothermia	Uh, probably education. You know for the surgical side because I think that we don’t get a lot of that in the training, we know it’s important but what steps, what checklists, we, I don’t think, well I haven’t. (S9) Information, just … Information, yeah knowledge, what works best, what’s the most economical, what’s readily available and … just what assists and then how patient outcomes at the end of the process, if you come out cold, is their stay in hospital another day, is their pain relief harder to deal with, is there, I’d like to just know those sorts of information so that I can use that information when I’m educating staff, new staff as well as older staff. (SS5)	11
	I know and understand the parameters for perioperative hypothermia	Right, well, I guess perioperative hypothermia refers to the patient’s body temperature being less than 36 degrees Celsius. Perioperative means it may be pre-op, intra-op ,or post-op. (A11)	2
	I understand how to monitor and prevent perioperative hypothermia	Warming blankets, hot dogs, warm clouds … Bair Hugger, all of those sort of interventions that we usually put in. Keeping the patient warm beforehand and on transport. (SS4)	3
Skills	The ability to monitor temperature is important in preventing perioperative hypothermia	To prevent it? To constantly be monitoring your patient, being aware of what their temperature is in theater. (PN1) And I think another thing that is very important is actually taking the preop temperature so that we know the baseline, where it is sitting. People sit in different temperatures … some high and some low, we cannot regulate everything, and we could end up with an even worse scenario or outcome. (AN3) So, knowing how to take someone’s temperature, knowing how to use the devices to keep them warm, that’s about it really, I think. I can’t think of anything else. (A11)	8
	I feel I have the skills necessary to manage and prevent perioperative hypothermia	My ability to perform the skills I would rate as high, my diligence and attendance to it is probably not quite as good as it should be at times. (A2) I’d say I’ve got basic skills … (PN1)	3
Social/Professional Role and Identity	I believe it is the responsibility of everyone in the operating room (including surgeons and nurses) to manage the patient’s temperature*	Every practitioner that sees the patient along their entire stay (is responsible for monitoring, managing and preventing perioperative hypothermia). (PN12) Uh I will just take you know, the usual, team effort approach. (S9) So, I think it is a multi-disciplinary responsibility, so it is surgeons, anesthetists, unit managers, educators, staff, all of those, yes. (8CF)	10
	It is predominantly the anesthetists‘ responsibility to manage the patient’s temperature*	Well, intraoperatively it’s the anesthetist, overall that’s a harder question to answer because the anesthetist has no control over many things outside the operating theater but I think at the end of the day it’s our role to keep all physiological variables as normal as we can and that’s one of them. (A2) Well I think the anesthetist has the primary role, the main role. Because we need to have a leadership role in theater to start off with. Obviously if the perioperative nurses and anesthetic technicians have a proactive attitude to it, it helps us but I would be taking the responsibility for the outcomes of the patients so, if they became hypothermic or had a complication, I would take that personally as my responsibility. (A11) Well the anesthetist is the one in the theater that is looking after that because they have the monitors there and they are able to tell us, and they’re monitoring the patients’ vitals and they can see that. They’ve also got the temperature probes, so they are basically taking on that responsibility and we can only assist them at that time, but once in recovery it’s the anesthetic, the recovery nurses and the team that have to monitor and keep the patients warm. (SS5)	10
	It is not a scrub scout nurses‘ responsibility to manage the patients‘ temperature	That’s not part of my role as a scrub/scout, I mean it is but its’ not my primary sort of thing, that is usually anesthetics or the anesthetist and stuff, but … I mean I still have a basic understanding of what we need to do, like keep them covered and all of that sort of stuff from uni even though I haven’t had direct training in it. Does that make sense? (SS4)	1
	It is/is not my role to manage the patients‘ temperature	My role is a bit of a, you know, you have … pretty primary. I am dealing with my patient face to face and so I feel as if I am responsible for their warmth and their care until they are handed over to the surgeon and the anesthetist in the theater. But I certainly will take them on as my responsibility from the time I pick them up until I get them into the anesthetic room and then into theater, so it is a primary carer at that time. (SS5) Um, yeah I can sort of passively delegate that pretty well, thank you. (S9)	6
Beliefs about capabilities	It is easy/challenging to manage my patient’s temperature*	Yeah, it’s easy, doable, achievable. (PN1) I would say it is easy for me to implement practices that would minimize hypothermia. There are times when during the procedure there is very little that you can do and then you need to spend some time to improve it at the end of the surgical procedure. (A2) Well, there’s certain surgical or cases that are more challenging than others for example prolonged laparoscopic cases can be very hard to keep the patient’s temperature up and major plastics cases like tram flaps. (A11)	12
	My ability to manage the patients‘ temperature effectively is limited by factors beyond my control*	Well there’s, sometimes if I’m scrubbed or if I’m … sometimes if I’m not going to be able to … like I can’t … (SS4) Right, so, some things are easy, some things are difficult. So, the things that I have direct control over are easy to do. So I can quite easily warm a patient in the induction bay and I can warm a patient in theater, so, and I can, and in recovery too, if I suggest to the recovery nurse that we need to warm this patient then they will do it, there is no barrier there. The main … probably the only time I don’t have much control is in the preoperative area where the nurses are admitting the patients and taking their obs and then letting them sit and wait for their surgery. (A11)	9
	I do/do not feel confident in my ability to manage patient’s temperature*	I wouldn’t say I am an expert at it (monitoring, managing & preventing perioperative hypothermia). (PN1)	2
Optimism	We are managing perioperative hypothermia effectively	Most of the time we are doing pretty well and the staff, with scrub/scout, recovery and holding bay, everyone, anesthetic … quite well. (AN3) I think as a department we’re pretty good, like a team. (SN4)	8
Beliefs about consequences	If perioperative hypothermia is not managed, this can result in adverse health outcomes*	Patient outcomes, poor patient outcomes, post op care and their recovery (will happen if we do not monitor, manage and prevent perioperative hypothermia). (PN1) Adverse outcomes, patient outcomes. It’s as simple as that. (CN7) … it contributes to all manner of adverse patient outcomes ranging from altered coagulation to impaired wound healing, patient discomfort, shivering, acidosis, eventually if you let people get cold enough, arrhythmias and even death. (A2)	12
	I/we do not know the impact of our efforts in preventing perioperative hypothermia*	I don’t really have any, it is a bit hard in recovery because you don’t see them. I don’t know what the long-term consequences really are, but I would say I guess it would have something to do with their long-term recovery, how they recover. (PN1) I think just in general the management of hypothermia is improving, in my opinion, um, and that maybe … feedback to the staff would be helpful, yeah like if it was, I don’t know patients, like pre-op, intra-op and post-op temperatures were recorded for a day and then they came back to a staff member and said hey your patients were really warm what did you do or your patients were really cold, that would help change and implement better practice. That’s about it. Yeah, because once the patients out of the theater you very rarely ever see them again so you are not aware if they are cold or if they’ve had a DVT or when they’ve gone home. So yeah we ‘re so isolated that I think acknowledgement of your care would help people I guess. *(Prompt)* I think that would apply to everyone, like every area yeah, maybe not so much with the pediatrics but with adults and obstetrics I think it would be noteworthy. (AN6)	3
Reinforcement	Awareness of the adverse outcomes associated with perioperative hypothermia acts as an incentive to manage the condition.	Data. I think, if you present staff, especially well-educated staff, which we should all be, here, if you present staff saying this is the data that says if you keep this patient’s temperature at this level the outcomes this … his statistically significant outcomes are going to be this. For me, that’s what I would think is an incentive. (CN7) Or maybe feedback would be good. So, for example, we always hear about cold patients but maybe if you hear about all the patients that are warm maybe that would be helpful, yeah, um, yeah that’s about all I can think of. (AN6) … some feedback in terms of, you know, how it is going over time and if it is getting better or worse and I think that is really helpful for people. Whether you strictly incentivise it or not … is I think questionable, but just trying to keep everyone in the team on board. (S10)	8
Goals	Perioperative hypothermia prevention practices are (very) important	Yeah well, I know I feel it’s … important and a high priority, that’s why I have it there set up ready to go. (PN1) Oh, it’s probably on the top five, yep. (CF8) Um, yeah, it’s an important concept I think, it’s when we um, particularly when we are operating for long periods we need to pay attention to keeping the patient normothermic, so maybe 37 degrees or above, um, yep. (S9)	8
	Other goals are more important than preventing perioperative hypothermia	… I guess one time which makes it difficult is in emergencies, yeah so like um, both in obstetrics and in adults like, the last thing you actually get to is the warming and I think that’s just in the hierarchy of needs yeah, and you’re playing catch up. (AN6) Yeah, I would have said it gets a relatively low priority for most anesthetists and theater teams. There’s is a couple of anesthetists here who are really big about it but others who are less so and the rest of us are probably somewhere in the middle. (A2)	7
Memory, attention, and decision processes	My attention to perioperative hypothermia prevention could sometimes be improved	To be honest, if anything when it is really, really busy. Yeah, if you’ve got other issues going on like pain and nausea, that would kind of, I feel, takes my priority away from, yeah, looking at their temperature and managing that, so I guess, that would be it. (PN1) So I think it is a priority but there are so many other things going on with a complex case that it’s easy to miss. (S9)	8
	I remind others to implement hypothermia prevention	Sometimes pretty good, sometimes really don’t, don’t have any perception that the patient may be cold, or exposed or anything like that, yeah, and that’s alright, that’s just education as well, all we need to do is just bring their attention to it, just say did you, you know, do you see this patient’s got a lot skin there we are waiting for the scrub team to wash their hands, I think we could cover them up for a bit longer, keep them, you know, it’s just participation, communication, and participation amongst the team members. I don’t think it’s any particular barriers, it’s just education … thoughtfulness. (SN4)	4
	I/others have processes or rituals in place to remind me to implement practices to prevent or manage perioperative hypothermia	I have my tray, my bedside tray, set up with my thermometer closest to the patient with a pen and piece of paper ready to go and that kind of triggers me to do it and record it straight away when the patient comes out. (PN1) Um, so I guess rituals would be from the start when I get the patient I always get them a warm blanket, I give it to them to hold, um, as we walk down to the induction room, and then when we are in the induction room I‘ll get them onto the trolley, the blanket goes on and then the dressing gown goes over it and then for monitoring, I always put the temperature probe um, there’s a little um, tray on the anesthetic machine where all the airway devices are and I have one underneath with the top blue part showing so it’s like a trigger for me to see it to get it, yeah. (AN6)	7
	Paying attention to keeping the patient covered is very important	Ok, um, so, first of all is keeping the patient warm and covered, um, not necessarily always by a warm blanket, like in the induction room, but just so they don’t um, radiate the heat, then we intraoperatively, you know, have bair huggers, warm fluids, um, for a lot of joints um if the bottom of the patient is exposed we wrap their head in a blanket or a towel, yeah, and using our monitoring as well obviously. (AN6)	2
Environmental context and resources	The availability/non-availability of resources affects my practice in preventing hypothermia*	The resources are all there ready to go and readily available. (PN1) They are … they are available here. Well, I see everything that is possible, maybe there’s something else that we can be doing – I don’t know. But everything that I know of, is available to us here. (SN5)	11
	Availability/non-availability of checklists and documentation affects my ability to record temperature and prevent hypothermia	If they’re concerned about it they should have a … something on the pre-op checklist, if they want us to initiate something preoperatively …because that’s basically our one piece of paper pre-op – and then during will have to be up to the anesthetist, and then post-op, I think it’s well done but if there’s improvement there then it should be identified and fixed on the PACU post-op form. (CN7) Again, some guidelines as to how to manage that and then some sort of established monitoring process and then someone who owns that, takes responsibility for that would be really helpful. (S10)	7
	Control of ambient temperature impacts upon our management of perioperative hypothermia	Fix the air con, make it more adjustable, which they are doing now, but we have not seen the result of that yet. (AN3) Well I guess as I eluded to before, the individual control of operating theater temperature is unreliable and my understanding is that’s not limited to here – that that’s a result of the way the buildings are engineered, but a smarter building design with better ability to control the temperature more precisely would be useful particularly in a place that does pediatric anesthesia as well. Yes … can’t think of any other environmental … we could just open the windows on a day like today! Oh, that’s right – we don’t have windows. (A2)	6
	The environmental layout and organization assists/impedes my ability to prevent perioperative hypothermia	So, intraoperatively and postoperatively there is good support. Preoperatively I don’t think it is existing, and *I* think the problem is that it is hard to come up with rock solid effort and space. Look here it is, because lots of hospitals do their little pilots and you get different results from different hospitals, some say yay and others say no. Because our layout is not that easy, people just sit and watch morning TV, while they wait. (CF8)	5
	There is a lack of communication in the workplace regarding perioperative hypothermia	I think generally people are pretty good with it, I think it is just that there’s not a lot of communication about it. I think that’s the biggest thing. I don’t think it is necessarily ignored by anyone, it’s just that there’s not really a lot of communication *…* (SN4)	1
	I have concerns regarding the accuracy of the temperature monitoring devices that are available to us	… temperature monitoring and would probably better in recovery if we had monitors that monitor … thermometers that were accurate, very inaccurate sometimes and they call them random number generators from time to time, so that’s a little bit tricky. (SN5)	3
Social influence	Other team members support/impede my temperature management practices	There is some resistance from surgeons to some warming methods so some, especially orthopedic surgeons, they worry about increasing infection rates so they don’t like using a device like the Warm Cloud. Most surgeons don’t like us turning the forced air warmers on before they have finished prepping and draping which can take a long time and the prepping is one of the things that can make the patient cold very quickly. So, there’s a couple of barriers there, but otherwise it is pretty straight forward. (A11) So, ah, I guess either awareness to start off with, I think it’s a team effort, so you know a lot of times surgeons, we probably don’t think about it as much as we should but you do see nursing staff a lot more active, anesthetists a lot more active in that part. (S9) *Oh, I think people are very supportive once you initiate it and there’s some anesthetist particularly advocative of preoperative warming*. (CN7)	12

**Abbreviations:** DVT, deep vein thrombosis; PACU, Post-Anesthetic Care Unit; TDF, Theoretical Domains Network.

**Table 2:** TDF domains less likely to inform interventions to improve perioperative hypothermia prevention practices

TDF domain	Specific belief	Example quotations	Frequency out of 12
Intention	I/we intend to implement practices to prevent perioperative hypothermia	Oh, it’s something I try and think about every time I anesthetize a patient. (A2)	4
Emotion	I do not feel any strong emotion regarding prevention of perioperative hypothermia	Not really. I don’t have any emotion attached to that. (S9)	1
I feel positive towards using interventions to prevent perioperative hypothermia	Totally. Yeah, I am all for it. So, like my motivation is tops, yeah. But, I haven’t really done a proper formal sort of analysis but I think all staff would agree that it is an important thing and would be on top of that as well. (CF8)	1
Behavioral regulation	We need to monitor and plan to implement perioperative hypothermia prevention practices	… I think we all need to be so much more mindful of positioning and you know, Bair Huggers and warm fluids and so forth. (S10)	4
If temperature management practice were standardized within the hospital, I would be more likely to manage perioperative hypothermia	If there was some sort of, same sort of thing applied, you know if it was like, when the patient comes in, make sure they have a warm blanket on during the procedure, make sure … I don’t know, if they have a temp probe catheter and all that sort of stuff, monitoring that like there was … which I mean, I guess … (SN4)	4

**Table 3:** COM-B model and TDF domains: suggested intervention strategies to improve perioperative hypothermia prevention

TDF domain	COM-B	Intervention function	BCT taxonomy	Individual BCT	Strategy example
Knowledge	C (Psychological)	Education	Goals and planning Feedback and monitoring Associations	Information about consequences Feedback on behavior and outcome of behavior Prompts/cues Self-monitoring	Audit and feedback of key prevention activities (temperature monitoring, warming – *behavior*) and rates of hypothermia (*outcomes*) Reminders of prevention activities in all key clinical areas; computerized reminders
Memory, attention, and decision processes	C (Psychological)	Training	Shaping knowledge	Demonstration of behavior Feedback on behavior and outcome of behavior Self-monitoring Behavioral practice/rehearsal	Education: when to monitor; how to monitor; when and how to warm; how to document Education: scenarios that allow for practice and feedback
		Environmental restructuring	Associations Antecedents	Adding objects to the environment Prompts/cues Restructuring of physical environment	Reminders (see above) Ensure monitoring is visibly obvious with reminders
Enablement	Goals and planning Feedback and monitoring Antecedents	Social support (unspecified and/or practical) Goal-setting (behavior and/or outcome) Adding objects to the environment Problem-solving Action planning Self-monitoring of behavior Restructuring of physical environment Review behavior and outcome goal(s)	Identified champion to monitor and provide encouragement Monthly agreed goals (based on audit and feedback) Reminders and equipment (see above) Provide hypothermia prevention pathway
Skills	C (Physical)	Training	Shaping knowledge Feedback and monitoring	Demonstration of behavior Feedback on behavior and outcome of behavior Self-monitoring Behavioral practice/rehearsal	Education (see above) Audit and feedback (see above)
Social/Professional Role and Identity	M (reflective)	Education	Shaping knowledge Feedback and monitoring Associations	Information about consequences Feedback on behavior and outcome of behavior Prompts/cues Self-monitoring	Make information available about consequences of not monitoring, warming, and outcomes of condition Prompts (see above) Audit and feedback (see above)
		Persuasion	Comparison of outcomes Natural consequences Feedback and monitoring	Credible source Information about social, environmental and health consequences Feedback on behavior and outcome of behavior	“High status” professional to provide information/education re importance of prevention Patients’ perspective of condition (and consequences) Audit and feedback (see above)
Modeling	Comparison of behavior	Demonstration of behavior	Provide demonstration of prevention pathway (ie, film/poster)
Beliefs about capabilities	M (reflective)	Education	Shaping knowledge Natural consequences Feedback and monitoring Associations	Information about consequences Feedback on behavior and outcome of behavior Prompts/cues Self-monitoring	Make information available about consequences of not monitoring, warming and outcomes of condition (as above) Prompts (see above) Audit and feedback (see above)
		Persuasion	Comparison of outcomes Natural consequences Feedback and monitoring	Credible source Information about social, environmental, and health consequences Feedback on behavior and outcome of behavior	High status’ professional to provide information (as above) Patients’ perspective of condition (and consequences) Audit and feedback (see above)
		Modeling	Comparison of behavior	Demonstration of behavior	Provide demonstration of prevention pathway (ie, film/poster)
		Enablement	Social support Goals and planning Antecedents Goals and planning Feedback and monitoring	Social support (unspecified and/or practical) Goal-setting (behavior and/or outcome) Adding objects to the environment Problem-solving Action planning Self-monitoring of behavior Restructuring of physical environment Review behavior and outcome goal(s)	Identified champion to monitor and provide encouragement Monthly agreed goals (based on audit and feedback) Reminders and equipment (see above) Provide hypothermia prevention pathway
Optimism	M (reflective)	Education	Shaping knowledge Feedback and monitoring Associations	Information about consequences Feedback on behavior and outcome of behavior Prompts/cues Self-monitoring	Make information available (as above) Prompts (see above) Audit and feedback (see above)
		Persuasion	Comparison of outcomes Natural consequences Feedback and monitoring	Credible source Information about social, environmental, and health consequences Feedback on behavior and outcome of behavior	High status’ professional to provide information (as above) Patients’ perspective of condition (and consequences) Audit and feedback (see above)
		Modeling	Comparison of behavior	Demonstration of behavior	Provide demonstration of prevention pathway (ie, film/poster)
		Enablement	Social support Goals and planning Antecedents Goals nd planning Feedback and monitoring	Social support (unspecified and/or practical) Goal-setting (behavior and/or outcome) Adding objects to the environment Problem-solving Action planning Self-monitoring of behavior Restructuring of physical environment Review behavior and outcome goal(s)	Identified champion to monitor and provide encouragement Monthly agreed goals (based on audit and feedback) Reminders and equipment (see above) Provide hypothermia prevention pathway
Beliefs about consequences	M (reflective)	Education	Goals and planning Feedback and monitoring Associations	Information about consequences Feedback on behavior and outcome of behavior Prompts/cues Self-monitoring	Make information available (as above) Prompts (see above) Audit and feedback (see above)
		Persuasion	Comparison of outcomes Natural consequences Feedback and monitoring	Credible source Information about social, environmental, and health consequences Feedback on behavior and outcome of behavior	High status’ professional to provide information (as above) Patients’ perspective of condition (and consequences) Audit and feedback (see above)
		Modeling	Comparison of behavior	Demonstration of behavior	Provide demonstration of prevention pathway (ie, film/poster)
Goals	M (reflective)	Education	Goals and planning Feedback and monitoring Associations	Information about consequences Feedback on behavior and outcome of behavior Prompts/cues Self-monitoring	Make information available (as above) Prompts (see above) Audit and feedback (see above)
		Persuasion	Comparison of outcomes Natural consequences Feedback and monitoring	Credible source Information about social, environmental, and health consequences Feedback on behavior and outcome of behavior	High status’ professional to provide information (as above) Patients’ perspective of condition (and consequences) Audit and feedback (see above)
		Incentivization	Feedback and monitoring	Feedback on behavior and outcome of behavior Monitoring of behavior, and/or outcomes of behavior, by others, without evidence of feedback Self-monitoring of behavior	Audit and feedback (see above)
		Coercion	Feedback and monitoring	Feedback on behavior and outcome of behavior Monitoring of behavior, and/or outcomes of behavior, by others, without evidence of feedback Self-monitoring of behavior	Audit and feedback (see above)
		Modeling	Comparison of behavior	Demonstration of behavior	Provide demonstration of prevention pathway (ie, film/poster)
		Enablement	Social support Goals and planning Antecedents Goals nd planning Feedback and monitoring	Social support (unspecified and/or practical) Goal-setting (behavior and/or outcome) Adding objects to the environment Problem-solving Action planning Self-monitoring of behavior Restructuring of physical environment Review behavior and outcome goal(s)	Identified champion to monitor and provide encouragement Monthly agreed goals (based on audit and feedback) Reminders and equipment (see above) Provide hypothermia prevention pathway
Reinforcement	M (automatic)	Training	Shaping knowledge Feedback and monitoring	Demonstration of behavior Feedback on behavior and outcome of behavior Self-monitoring Behavioral practice/rehearsal	Education: when to monitor; how to monitor; when and how to warm; how to document Education: scenarios that allow for practice and feedback
		Incentivization	Feedback and monitoring	Feedback on behavior and outcome of behavior Monitoring of behavior, and/or outcomes of behavior, by others, without evidence of feedback Self-monitoring of behavior	Audit and feedback (see above)
		Coercion	Feedbackand monitoring	Feedback on behavior and outcome of behavior Monitoring of behavior, and/or outcomes of behavior, by others, without evidence of feedback Self-monitoring of behavior	Audit and feedback (see above)
		Environmental restructuring	Associations Antecedents	Adding objects to the environment Prompts/cues Restructuring of physical environment	Prompts (see above) Provide hypothermia prevention pathway
Environmental context and resources	O (physical)	Training	Shaping knowledge Feedback and monitoring	Demonstration of behavior Feedback on behavior and outcome of behavior Self-monitoring Behavioral practice/rehearsal	Education: when to monitor; how to monitor; when and how to warm; how to document Education: scenarios that allow for practice and feedback
		Restriction	No BCTs	No BCTs	
		Environmental restructuring	Associations Antecedents	Adding objects to the environment Prompts/cues Restructuring of physical environment	Prompts (see above) Provide hypothermia prevention pathway
		Enablement	Social support Goals and planning Antecedents Goals and planning Feedback and monitoring	Social support (unspecified and/or practical) Goal-setting (behavior and/or outcome) Adding objects to the environment Problem-solving Action planning Self-monitoring of behavior Restructuring of physical environment Review behavior & outcome goal(s)	Identified champion to monitor and provide encouragement Monthly agreed goals (based on audit and feedback) Reminders and equipment (see above) Provide hypothermia prevention pathway
Social influence	O (social)	Restriction	No BCTs	No BCTs	
		Environmental restructuring	Associations Antecedents	Adding objects to the environment Prompts/cues Restructuring of physical environment	Prompts (see above) Provide hypothermia prevention pathway
		Modeling	Comparison of behavior	Demonstration of behavior	Provide demonstration of prevention pathway (ie, film/poster)
		Enablement	Social support Goals and planning Antecedents Goals and planning Feedback and monitoring	Social support (unspecified and/or practical) Goal-setting (behavior and/or outcome) Adding objects to the environment Problem-solving Action planning Self-monitoring of behavior Restructuring of physical environment Review behavior & outcome goal(s)	Identified champion to monitor and provide encouragement Monthly agreed goals (based on audit and feedback) Reminders and equipment (see above) Provide hypothermia prevention pathway

**Abbreviations:** BCT, Behavior Change Theory; TDF, Theoretical Domains Network.

## Results

Twelve participants (two anesthetic nurses, two scrub-scout nurses, two post-anesthetic care unit nurses, one clinical nurse facilitator, one nurse manager, two anesthetists, two surgeons) took part in this study. This corresponded to eight females and four males across the multidisciplinary groups. Participant’s years of experience in their relevant perioperative specialty ranged from 2 to 30 years. The duration of interviews ranged from 9.52 to 28.15 minutes (median duration=18.3 minutes). Data saturation was assessed as occurring after 12 interviews.

### Key domains

Eleven theoretical domains were identified as relevant to the implementation of perioperative hypothermia prevention across interviews with the multidisciplinary participants: knowledge; skills; social/professional role and identity; beliefs about capabilities; optimism; beliefs about consequences; reinforcement; goals; memory, attention, and decision processes; environmental context and resources; and social influence (see Table 1).

It was evident that both nursing and medical participants were unsure as to the existence of guidelines for prevention of perioperative hypothermia (Knowledge). Some participants expressed that they did not know of any, whilst others expressed a belief that they must be in existence, although they could not identify them. The existence of the UK-based National Institute for Health and Care Excellence guidelines was recognized, but whether Australian guidelines were in existence was a source of confusion.^1^ Although prevention of the condition was recognized as important (Goals), a general lack of knowledge regarding the condition was evident and recognized by the individual participants, both in relation to their own knowledge, but also that of their colleagues (Knowledge). Whilst anesthetists in this study were confident in their knowledge of the condition, surgeons interviewed were noted to be less so. Nurses with greater years of experience in the speciality expressed a greater knowledge of the condition. Nursing participants noted that those who felt they had formal knowledge of the condition, including the characteristics and preventative strategies, had acquired this via extra study, attendance at conference workshops, and/or further education. The need for further education on the condition was widely cited across the participant groups, expressed both as a need by individuals themselves, but also as a strategy for colleagues to improve their knowledge of the condition (Knowledge).

The practice of monitoring the patient was consistently identified as being highly important in perioperative hypothermia prevention, by all stakeholders (Skills). This included ensuring a baseline temperature was recorded. While no concerns over possessing the skill to monitor temperature were expressed, and the availability of devices to monitor temperature was not reported as a barrier, concerns were reported over the accuracy of available devices (Environmental Context and Resources), especially by anesthetic and PACU nursing participants. Furthermore, the inability to control ambient temperature was acknowledged across participant groups as being an environmental factor that impacts upon the ability to manage perioperative hypothermia. Although it was felt that that concrete resources, in terms of devices and monitors, were available, it was identified that checklists, documentation, and guidelines would facilitate the implementation of practices to prevent perioperative hypothermia.

Discrepancies were evident in participants’ perceptions of whom is ultimately responsible for perioperative hypothermia prevention. Whilst participants widely expressed they believed this was a team effort, and that all healthcare practitioners involved in the care of the perioperative patients should take responsibility for prevention, the central position of the anesthetist in temperature-related decision-making was emphasized (Social/Professional Role and Identity). This was particularly so in relation to intraoperative monitoring, where it was felt that the anesthetist is in charge of this, so other team members have less of a role at this stage. In particular, the lesser involvement of scrub scout nurses was highlighted by both scrub scout nurses themselves, as well as the participating anesthetists. A perceived inability or lessened capacity to contribute to hypothermia prevention was evident in the beliefs of scrub scout nurses in this respect (Professional Role and Identity).

To some degree, the prevention of perioperative hypothermia was identified as being achievable. However, it was also recognized that barriers persist in prevention, and that prevention is hindered by factors outside of the perioperative healthcare practitioners’ control (Beliefs about capabilities). In this regard, it was recognized that the patients’ health status and outcomes (including temperature) at each stage of the perioperative pathway are influenced by the preceding stage. Participants recognized that they may have little control over the actions taken in the preceding stage that may result in patients in their care already having experienced temperature decline. For example, an anesthetic nursing staff participant noted that the practices of preoperative staff (including whether patients lost heat in the waiting area) influenced whether the patient was hypothermic in the anesthetic phase of care. The impact of surgical factors that directly influence temperature decline and which may, or may not, be controlled for were also highlighted, in particular by the anesthetic medical participants (Beliefs about capabilities). Nonetheless, and conversely, a sense of optimism prevailed in that participants believed the department was doing well in the prevention of perioperative hypothermia, and that colleagues were motivated to prevent the condition (Optimism).

The recognition that perioperative hypothermia would result in adverse patient outcomes was widespread across the participants, however anesthetic medical staff were most able to specifically identify associated outcomes. It was evident that some nursing participants understood that the condition was detrimental, yet were unable to communicate further as to the specific, adverse associated outcomes. It was also expressed that both medical and nursing participants felt that the outcomes extended beyond the realm of care that they were directly associated with, and that they were not aware of the adverse outcomes as they did not see them in their phase of care (Beliefs about consequences). This also meant that they did not see the positive aspects of the preventative care that they may have enacted. It was felt that reporting of the adverse consequences associated with perioperative hypothermia would act as an incentive for healthcare professionals to improve their preventative practices – as would reporting of the positive outcomes that might arise from proactive preventative care (Reinforcement).

Although it was widely agreed that perioperative hypothermia prevention is important, it was also clear that other goals were seen as more important in the “hierarchy of needs” – acknowledging the acute nature of surgical care – and, therefore, hypothermia prevention practices can shift down the list of priorities within the perioperative department (Goals). Whilst it is important, all participant groups noted that other clinical issues can affect the attention that is paid to keeping patients warm and implementing temperature monitoring (Memory, Attention, and Decision Processes), acknowledging that their individual attention to prevention can sometimes be improved. The existence of individual rituals or routines to serve as a reminder to implement preventative practices (such as the application of a warming blanket, or monitoring of temperature) were reported, particularly by anesthetic and PACU nurses. Some participants also felt they regularly reminded colleagues to enact practices to prevent heat loss, including reminders of the importance of keeping patients covered as much as possible.

All participants highlighted the impact that other team members had on either supporting or impeding their perioperative hypothermia prevention practices (Social Influences). The assertion that most colleagues are supportive appears to align with the optimism expressed by some participants, however others expressed a conflicting belief that resistance to implementation of preventative practice (particularly warming) was experienced. Reluctance of surgeons in relation to application of forced air warming, particularly in the orthopedic speciality, was reported by anesthetists, however not by surgeons themselves. Nonetheless, the importance of social influence was evident across all stakeholder groups.

### Domains reported as less relevant

Three domains were assessed as less relevant; intention; emotion; and behavioral regulation (see Table 2). Intention to implement perioperative hypothermia prevention practices was evident in four of the 12 interviews, and in these interviews individual intention to implement, as well as the perception of team members’ intention, was reported. Across the multidisciplinary team, it was reported that strong emotions were not associated with perioperative hypothermia prevention, however one nursing participant expressed a strong motivation (Emotion). The perceived need to regulate behavior was not identified across the interviews (Behavioral Regulation): in those interviews where beliefs relevant to this domain were evident, a need for planning to implement prevention practices was expressed.

### Mapping to COM-B model and identification of potential intervention strategies

The strategies likely to improve the implementation of perioperative hypothermia prevention identified via mapping to the COM-B model and BCTs are audit and feedback, reminders and prompts, education (including information delivered by a “high status” healthcare professional), the use of an identified “champion” to drive improvements, care pathways, and monthly agreed goal setting (see Table S3). The restriction intervention label has no corresponding BCT. The identified issues regarding the accuracy of available temperature monitoring devices in the department appears to correspond to this category as a factor which limits implementation of perioperative hypothermia (and is worthy of attention), but which is not related to behavior.

## Discussion

This study is, to our knowledge, the first that explores the barriers and enablers to perioperative hypothermia prevention from the perspective of key healthcare professionals across the perioperative department. Furthermore, a strength of this study is the generation of an understanding of the behaviors influencing perioperative hypothermia prevention via the use of the TDF as a theory-driven approach. This enabled us to identify 11 key theoretical domains of behavior that influence the uptake of perioperative hypothermia practices: knowledge; skills; social/professional role and identity; beliefs about capabilities; optimism; beliefs about consequences; reinforcement; goals; memory, attention, and decision processes; environmental context and resources; and social influence.^17^,^18^ Consideration of these domains alongside the BCW has allowed for identification of suggested intervention strategies to improve uptake of key perioperative hypothermia prevention activities in clinical practice, including temperature measurement and implementation of warming strategies.^26^

A key barrier related to the confusion and uncertainty around the existence of perioperative hypothermia guidelines, which was particularly evident across all interviews, and participant groups. While the UK’s National Institute for Health and Care Excellence (NICE) guidelines^1^ were mentioned by one anesthetist, participants were unsure if there were any Australian guidelines. The Australian and New Zealand College of Anesthetists (ANZCA) published an audit tool for perioperative hypothermia in 2014^28^ (based upon the UK Royal College of Anaesthetist audit tool^29^), and it is also worth noting that just a few months after data collection (in May 2018) the Australian College of Perioperative Nurses (ACORN) published the first nurse-led guidance on the topic.^11^ The lack of familiarity with perioperative hypothermia guidelines found in this study mirrors previous findings that anesthetists are unfamiliar with temperature measurement guidelines,^16^ and that nursing knowledge of hypothermia is lacking.^30^–^32^ While the development of the ACORN Standard^11^ represents progress in regards to perioperative hypothermia prevention in Australia, it is important to recognize that this is a nursing-focused guideline, and the propensity for acceptance of this new, nursing-led guidance by medical perioperative staff cannot be predicted. The provision of perioperative hypothermia guidance in the Australian medical perioperative field, in addition to the clinical audit tool,^28^ would be a valuable next step: the ability for guideline developers in both the nursing and medical fields to work together, to ensure that both nursing and medical-led perioperative hypothermia guidelines are congruent, can be seen as vital.

Another clear finding from this study was participants’ self-identified lack of knowledge, and a need for education regarding perioperative hypothermia in terms of the etiology of the condition itself, but also in regards to best practice recommendations for prevention. While both nursing and medical participants recognized that perioperative hypothermia was detrimental, depth of understanding of the physiology, and associated outcomes of the condition was variable. This was particularly an issue for the less experienced nursing staff, with some confusion of the difference between perioperative hypothermia and malignant hyperthermia – very different conditions – evident. Those nursing staff who expressed a greater confidence in their understanding felt they had gained this through either formal study or attending extra education external to the institution; however, even experienced nurses voiced a desire for additional education on the condition itself and prevention, which is reflective of findings from earlier research that even experienced perioperative nurses experienced knowledge deficits regarding the definitions of hypothermia and normothermia.^30^ This finding also supports the assertion of researchers who have evaluated nursing knowledge of hypothermia, that there is a need for ongoing education regarding perioperative hypothermia temperature cut-offs and temperature values.^31^ Based on the findings of this and other studies, it seems sensible that perioperative hypothermia prevention education should be made available to all members of the perioperative multidisciplinary team.^32^

While, on one hand, it was acknowledged by all participants that prevention of perioperative hypothermia is a team responsibility, conversely, the belief that the anesthetist should assume ultimate responsibility was also clearly expressed – a responsibility which the anesthetists in Boet et al’s^16^ study also identified. This appeared to be a role that the anesthetist participants were accepting of to a degree; however it was also acknowledged that, outside of the operating theatre itself, it is less easy for anesthetists to influence factors that can contribute to heat loss. For instance, in PACU, it was noted that it is clearly the PACU nurses’ role to monitor patients and manage hypothermia. The input of multidisciplinary staff at every point of the multi-staged perioperative pathway was acknowledged, as was the influence that care at each point can have on temperature decline in the next stage. In this regard, the impact that other team members, including at preceding stages of care, can have on individuals’ hypothermia prevention practices was recognized as both a detrimental and/or facilitative factor. Therefore, any initiatives aiming to improve perioperative hypothermia need to target practice at each stage, and all multidisciplinary groups involved with the care of patients in the perioperative area. This may serve to improve team cohesion in prevention across the perioperative pathway. This assertion is supported by earlier initiatives which have sought to facilitate implementation of guidelines to improve prevention of perioperative hypothermia and found that multidisciplinary buy-in to any initiative addressing perioperative temperature management practices is vital.^7^ Previous studies have also found, amongst anesthetists, a lack of importance attached to perioperative temperature monitoring.^33^,^34^ As regular and consistent temperature monitoring is a cornerstone of perioperative hypothermia prevention, this highlights the importance of prevention not relying upon the actions of one professional group.

The importance of preventing perioperative hypothermia was recognized across all participant groups; however, the existence of conflicting priorities that impacted upon implementation of preventative activities was widely reported, which is congruent with existing reports in the literature.^30^,^31^ It was suggested that reminders to bring attention back to perioperative hypothermia would be valuable in this regard. Again, in the context of perioperative hypothermia prevention, it seems reasonable that a reminder system would need to target all multidisciplinary team members at key stages of the perioperative pathway. Arditi et al’s^35^ systematic review highlighted that, although (computerized) reminders alone are likely to improve compliance with guidelines themselves, the actual impact on patient outcomes remains uncertain. Consideration as to how reminders are delivered – whether these are computerized, paper-based, or via other means – is also required^35^ in the context of perioperative hypothermia.

While sufficient availability of equipment, in terms of adequate numbers of warming devices or monitoring devices, was identified by participants, environmental factors related to lack of control of air conditioning and ambient temperature, departmental layout, and accuracy of available temperature monitoring devices were highlighted as a barrier to implementing practices to prevent perioperative hypothermia. This lack of confidence in preventing the condition due to environmental influences – and social influences (as discussed above) – aligns with findings in previous research^16^ that organizational and cultural context impacts upon practice. This is also reflected in the beliefs expressed by participants in our study that the ability to manage temperature is limited by factors beyond the individuals’ control. This is unsurprising given that the call for reliable and accurate temperature measurement devices, and increased control over environmental factors such as ambient temperature, is widely noted in the existing literature.^30^,^36^,^37^ Ensuring that accurate monitoring devices are available, and that the environmental conditions support prevention of heat loss can be considered prerequisites for perioperative hypothermia prevention. These factors cannot be attributed to behavioral influences in themselves, but can influence healthcare professionals’ perception of capabilities to prevent hypothermia.

Providing feedback to the team on patient outcomes specifically related to perioperative hypothermia and temperature measurement may facilitate improved practice via two mechanisms. Firstly, feedback on patient outcomes may assist those staff who identified a knowledge deficit in relation to the condition itself, and what the condition means for patient outcomes. Secondly, the nature of the perioperative area means that, in many instances, perioperative team members do not see the impact of their care beyond their immediate area. The use of audit and feedback has been suggested as a strategy for influencing perioperative hypothermia prevention practice, both in Australia^7^,^38^ and also in Canada where the use of benchmarked or ranked feedback against no feedback has been assessed, finding no difference in feedback versus no feedback.^39^ Existing recommendations that audit and feedback interventions need to be carefully considered in conjunction with other interventions – balanced with cost – seem prudent.^40^

Given the complex, multi-phased and multi-disciplinary nature of perioperative hypothermia prevention, it seems sensible that future interventions target all members of the perioperative team,^32^ and that training initiatives should be team-based rather than purely targeted at an individual level. Such training initiatives should aim to improve baseline knowledge of the underlying condition (perioperative hypothermia) as well as the preventative strategies recommended by guidelines. Based on the findings of this and other studies, it seems that multimodal or bundled strategies^39^ to improve implementation of perioperative hypothermia prevention need to be considered. The interventions suggested by this study, developed using the TDF and underpinned by the BCW, need to be assessed for affordability, practicality, effectiveness and cost-effectiveness, acceptability, safety, and equity using the APEASE criteria proposed by Michie et al.^26^ Input from the multidisciplinary perioperative team will be vital in the ultimate selection of these implementation strategies.

### Strengths and limitations

While this study included nursing and medical team members, it did not include non-nursing or non-medical perioperative staff because no anesthetic or theatre technicians agreed to participate, despite efforts to recruit them. In particular, anesthetic technicians can be seen to have a role in preventing perioperative hypothermia, and so the absence of their perspectives in this study can be seen as a limitation. Even though anesthetic nurses (included in this study) and anesthetic technicians work to support the anesthetist interchangeably, there may be particular issues which are experienced by anesthetic technicians that are not recognized by nursing staff. In future studies, the inclusion of their perspectives would be valuable. It is also recognized that some participants expressed a particular interest in the topic of perioperative hypothermia, and, in this regard, there is potential that their beliefs are not representative of the wider perioperative team and those who did not elect to take part. It is also acknowledged that this study design assesses expressed beliefs, and does not assess practice or actions. This study was also conducted in one healthcare facility, and therefore generalizability to other healthcare facilities may be limited. However, a strength of this study is that it does appear to build on similar work conducted by Boet et al,^16^ as previously discussed, in taking a multidisciplinary approach to the wider issue of perioperative hypothermia prevention, rather than temperature monitoring alone. Furthermore, the utilization of behavior change theories to develop the suggested interventions can also be considered a strength.

## Conclusion

This study examined the barriers and enablers to perioperative hypothermia prevention, as perceived by the multidisciplinary team, eliciting a deeper understanding regarding perioperative temperature management practices. By utilizing the TDF, 11 theoretical domains that influence perioperative hypothermia prevention practices were identified and, by the application of behavior change theory, suggested intervention strategies were identified.

Through the application of behavior change theories, the key components of provision of education specifically related to perioperative hypothermia prevention, reminders/prompts, and audit and feedback were identified. These strategies should target the entire multidisciplinary team involved at all stages of the perioperative pathway to facilitate a cohesive approach to perioperative hypothermia prevention. However, only future feasibility studies will determine the acceptability of these interventions in specific health contexts, by the target clinicians.

## Data Availability

The datasets generated and/or analyzed during the current study are not publicly available, but are available from the corresponding author on reasonable request.

## Abbreviation list

BCT, Behavior Change Taxonomy; BCW, Behavior Change Wheel; HREC, Human Research Ethics Committee; PACU, Post Anesthetic Care Unit; TDF, Theoretical Domains Framework.

## Authors’ information

The researchers involved in the conduct of interviews (JM, AD) had a historical – but not current at the time of data collection – professional relationship with some of the participants. A full explanation of the study aims was provided during the recruitment and consent process (see Ethical considerations). The participants were aware that JM has conducted research on perioperative hypothermia.
